# Endoscopic hematoma removal of supratentorial intracerebral hemorrhage under local anesthesia reduces operative time compared to craniotomy

**DOI:** 10.1038/s41598-020-67456-x

**Published:** 2020-06-25

**Authors:** Masahito Katsuki, Yukinari Kakizawa, Akihiro Nishikawa, Yasunaga Yamamoto, Toshiya Uchiyama

**Affiliations:** Department of Neurosurgery, Suwa Red Cross Hospital, 5-11-50, Kogandori, Suwa, Nagano 981-0945 Japan

**Keywords:** Outcomes research, Neurology, Risk factors

## Abstract

The surgical efficacy for supratentorial intracerebral hemorrhage (ICH) remains unknown. We compared the advantages of the widely practiced endoscopic hematoma removal under local anesthesia with that of craniotomy under general anesthesia for ICH. We also focused on our novel operative concept of intentional hematoma leaving technique to avoid further damage to the brain. We retrospectively analyzed 134 consecutive patients (66 endoscopies and 68 craniotomies) who were surgically treated for supratentorial ICH. The characteristics of the 134 patients were as follows: The median (interquartile range) age was 73 (61–82) years. The median Glasgow Coma Scale scores at admission, on day 7, and the median modified Rankin Scale (mRS) score at 6 months were 10 (7–13), 13 (10–14), and 4 (3–5) respectively. The statistical comparison revealed there were no differences in GCS score on day seven between the endoscopy 13 (12–14) and craniotomy group 12 (9–14). No differences were observed in mRS scores at 6 months between the endoscopy 4 (2–5) and craniotomy group 4 (3–5). However, the patients treated with our technique tended to have favorable outcomes. Multivariate analysis revealed the operative time was significantly decreased in the endoscopy group compared to the craniotomy group (*p* < 0.001).

## Introduction

Spontaneous intracerebral hemorrhages (ICHs) are responsible for 10%-30% of all strokes and they remain a significant cause of all stroke-related mortality and morbidity^1,2^. ICH is a medical emergency with high fatality and disability rates. The median 30-day mortality rate after ICH is approximately 15–50%^3,4^ and only 20% of patients regain functional independence within three months after the ictus^5^.

Surgical hematoma removal and conservative therapy are the main treatments for ICH; however, the role of surgery for most patients with ICH remains controversial. Theoretically, surgical hematoma removal prevents herniation by reducing the intracranial pressure and decreasing the pathophysiological impact of the hematoma on surrounding tissue^2^. However, the effectiveness of surgery has been repeatedly evaluated^6,7^ and its benefits are still under discussion. The Surgical Trial in Intracerebral Haemorrhage (STICH) indicated that patients with spontaneous supratentorial ICH showed no overall benefit from the early surgery when compared to the initial conservative therapy. However, operative intervention occurred in 24% of patients in the initial conservative treatment group^6^. Therefore, the interpretation of these results is complicated. The STICH II trial confirmed that early surgery did not increase the rate of death or disability, 6 months postoperatively, and may have a small but clinically relevant survival advantage for patients with spontaneous superficial ICH without an intraventricular hemorrhage^7^. A recent report based primarily on the STICH II trial reported that only patients with a GCS 10–13 or a large ICH were likely to benefit from surgery^8^. However, the STICH and STICH II trials did not exhibit an overall comprehensive benefit for the functional outcome over medical therapy^9^. Furthermore, in these previous studies, almost all of the patients underwent craniotomy, therefore the benefit and efficacy of endoscopic hematoma removal remain unknown.

Endoscopic hematoma removal has become a widely popular practice as it reduces the operative time and invasiveness, and potentially improves the outcomes^9^. Compared to the stereotactic evacuation of ICH, hemostasis during the surgery can be easily achieved using a coagulator^10^. Besides, the endoscopic procedure is less invasive than craniotomy and can be performed with the patient under local anesthesia^10^. The effectiveness of endoscopic hematoma removal has been studied extensively. In 2016, phase II of the MISTIE trial (Minimally Invasive Surgery Plus Rt-PA for ICH Evacuation) demonstrated favorable preliminary results for the stereotactic aspiration and catheter drainage with a tissue plasminogen activator^11,12^. In addition, an endoscopic evacuation arm of MISTIE II, called the Intraoperative Stereotactic Computed Tomography-Guided Endoscopic Surgery (ICES), also demonstrated the safety and effectiveness of the chronic neurological outcome^13^. Recently, the MISTIE trial phase III demonstrated no functional benefit for the MISTIE procedure in selected patients; however, a subgroup analysis showed improvement of the 1-year outcomes in patients with an increased hematoma removal rate (≤ 15 mL residual hematoma after the surgery)^14^. In addition to these clinical studies, endoscopic evacuation methods are still being evaluated and undergoing improvements. There are currently two ongoing clinical trials; (1) the multicenter, single-arm feasibility study in the United States evaluating the Apollo system and the Artemis Device as minimally invasive surgical treatment options to treat patients with moderate-large volume (20–80 mL) supratentorial ICH within 24 h from the onset (INVEST: A Single Arm, Feasibility Study of Minimally Invasive Endoscopic Surgical Treatment With Apollo for Supratentorial ICH)^15^, and (2) a multicenter randomized clinical trial evaluating the effect of minimally invasive hematoma evacuation using the Artemis Neuro Evacuation Device with medical management compared to the best medical management alone (MIND: A Prospective, Multicenter Study of Artemis a Minimally Invasive Neuro Evacuation Device, in the Removal of Intracerebral Hemorrhage)^16^.

However, the efficacy of endoscopic surgery compared to craniotomy remains unclear due to the small number of reports^17–25^ and the heterogeneity of patients included in these studies. Only three meta-analyses (Table 1) have explored the effectiveness of the endoscopic procedures compared to craniotomy. Two of them concluded that endoscopic surgery decreased complications even though it did not decrease the mortality rates^26,27^. One of the meta-analysis, comparing both the endoscopy and stereotactic hematoma evacuation to craniotomy as well as the endoscopy to craniotomy and medication, demonstrated a significant benefit for the neurological function and mortality^28^. However, these meta-analyses used limited studies with a heterogeneous patient or selection bias; therefore, further studies without targeted patient selections are necessary. Here, we conducted a retrospective study that compared the effectiveness differences of endoscopic hematoma removal under local anesthesia compared to the traditional hematoma removal with craniotomy for treatment of patients with supratentorial ICH, in a real clinical setting.

**Table 1:** Comparison between our results and previous meta-analyses for supratentorial ICH^26–28^.

Year	Author	Included studies	Group	No of patients	Mean (Median) age	Mean (Median) GCS	Hematoma volume (mL)	Operative time (min)	Hematoma removal rate	Mortality	Complication	Long-term outcome	Summary
2018	Scaggiante	15 RCT	(Endo and stereo) vs cranio	441 vs 422	–	–	–	–	–	11% vs 19%	–	mRS 0–2 46% vs 30%	(Endo and stereo) increase the chance of being independent (OR, 0.44 [0.29–0.67]; *p* = 0.0002) (Endo and stereo) reduce mortality (OR, 0.56 [0.37–0.84]; *p* = 0.005)
2018	Scaggiante	15 RCT	Endo vs (cranio and medi)	183 vs 201	–	–	–	–	–	16% vs 29%	–	mRS 0–2 34% vs 17%	Endo increase the chance of being independent endoscopic surgery (OR, 0.40 [0.25–0.66]; *p* = 0.0003) Endo reduces mortality (OR, 0.37 [0.20–0.67]; *p* = 0.001)
2019	Zhao	3 RCT	Endo vs cranio	144 vs 151	64.4 vs 64.8	–	56.6 vs 51.0	–	–	6% vs 10%	24% vs 64%	–	Endo reduces mortality (RR = 0.58 [0.26–1.29]; *p* = 0.18) Endo lowers complications (RR = 0.37 [0.28–0.49]; *p* < 0.001)
2019	Nam	3 RCT	Endo vs cranio	144 vs 145	64.4 vs 64.1	9.17 vs 9.26	56.6 vs 51.0	103.4 vs 205.7	85.2% vs 78.2%	6% vs 10%	24% vs 64%	–	Endo reduces the mortality (RR = 0.58 [0.26–1.29]; *p* = 0.18) Endo reduces complication rate (OR = 0.11 [0.06–0.20]; *p* < 0.00001)
2020	Our study	Retrospective	Endo vs cranio	66 vs 68	73 vs 73	10 vs 9	104 vs 120	63 vs 105	98% vs 98%	10% vs 15%	24% vs 64%	mRS 0–2 28% vs 22%	No difference in GCS score on day 7, mRS at 6 months, mortality, complication nor rebleeding. Endo reduces operative time (*p* < 0.001)

Results in previous reports by the mean; our results are shown by the median.

GCS, Glasgow Coma Scale; cranio, hematoma removal with craniotomy; endo, endoscopic hematoma removal; medi, medication; mRS, modified Rankin Scale; RCT, randomized controlled trial; stereo, stereotactic hematoma evacuation; –, not described in detail.

## Materials and methods

### Study population

We retrospectively investigated the medical records of 134 surgically-treated consecutive patients with supratentorial ICH from 2012 to 2019. Patients without the outcome data at 6 months were excluded. The diagnosis of ICH was based on clinical history and the presence of ICH on computed tomography (CT). The inclusion criteria for the study were as follows; (1) patients with intracerebral hemorrhage at the basal ganglia or subcortex, (2) patients designated for surgical treatment according to the Japanese Guidelines for the Management of Stroke 2015^29^ and 2009^30^ (described in detail in the *General management* section) and treated endoscopically, and (3) the interval between onset and hematoma removal was less than 24 h. The exclusion criteria were as follows; (1) ICHs due to the tumor, trauma, aneurysm, arteriovenous malformation, and hemorrhage after infarction, and (2) patients who had a thalamic or caudate head hemorrhage with an intraventricular hemorrhage treated by the flexible neuroendoscope to only remove the intraventricular hematoma. The Suwa Red Cross Hospital’s research ethics committee approved this study, and we gained written informed consent for this study from all of the patients, the legally authorized representative of the patients, or next of kin of the deceased patients. All methods were carried out in accordance with relevant guidelines and regulations (Declaration of Helsinki).

## General management

During admission and in the acute phase, patients were first administered nicardipine to maintain the normal systolic blood pressure at under 140 mmHg. The prothrombin time of patients undergoing anticoagulation therapy was normalized by the administration of vitamin K and/or fresh frozen plasma. Then, a surgical indication was made following the Japanese Guidelines for the Management of Stroke 2015^29^ and 2009^30^. Both versions describe same surgical indications, which are as follows: Patients with hematoma at the basal ganglia which was more than 30 mL, and who were neurologically deteriorating were designated for surgery. Patients with superficial lobar hemorrhage within 1 cm of the cortical surface and with disturbance of consciousness or moderate neurological deficits were also designated for surgery. Patients with a small hemorrhage and without severe neurological symptoms that could be treated by conservation or patients with cardiopulmonary arrest on arrival did not undergo any surgical treatment. Rehabilitation and nutritional support were started immediately after the operation and steps were undertaken to prevent and treat the complications. Antithrombotic agents were discontinued postoperatively for several days depending on the patients’ condition and comorbidities.

Hematoma removal with craniotomy was performed primarily from 2012 to 2013 and the endoscopic hematoma removal began in 2013. We gradually transitioned from craniotomy to endoscopic hematoma removal as a first-choice treatment between 2014 and 2015. During this period, patients who received antithrombotic drugs and displayed apparent extravasation on the contrast-enhanced CT image were likely to undergo a craniotomy. Since 2015, endoscopic procedures have been routinely performed in our hospital regardless of age, comorbidities, presence of antithrombotic drugs, and extravasation on the contrast-enhanced CT image. However, a craniotomy was still performed when the endoscope was unavailable due to reasons such as cleaning or the unavailability of the medical staff in the operating room (i.e., weekends and holidays).

## Endoscopic procedure

**Figure 1:**
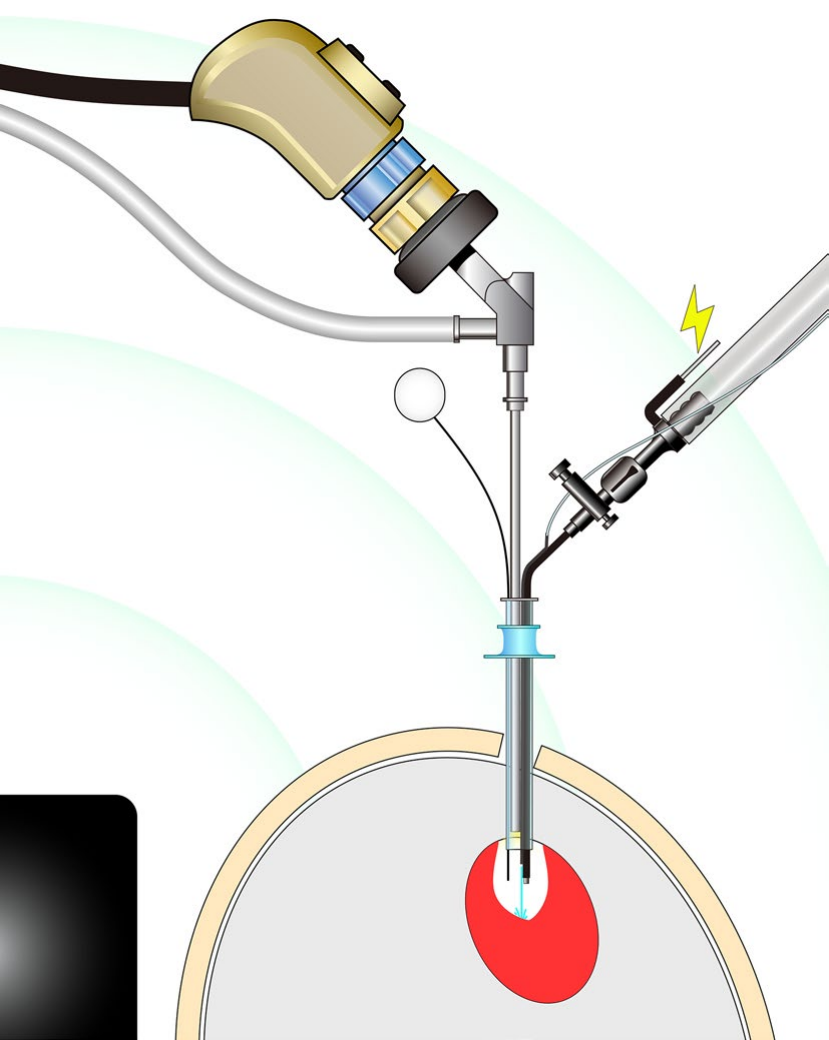
Schematic illustration of the endoscopic hematoma removal using the combined irrigation-coagulation suction cannula through the Neuroport, transparent sheath with the help of the electromagnetic neuronavigation. The multifunction cannula releases water from its tip for irrigation and is covered with a black electrical insulator, except for its tip and its proximal conductive part. This enables the cannula to coagulate the bleeding point at its tip by electrocoagulation supplied using a monopolar electro-cauterizer. The electromagnetic neuronavigation allows for frameless, pinless image-guided endoscopic hematoma removal under local anesthesia. The black object is the electromagnetic field emitter, and the green arcs imitate the electromagnetic field. The electromagnetic flexible navigation pointer is inserted through the NeuroPort and identifies the area that we are endoscopically manipulating.

We performed endoscopic hematoma removal under local anesthesia. We do not usually perform this procedure under general anesthesia due to the risks of general anesthesia and the time to start the surgery. We simultaneously also prepared for conversion to craniotomy under general anesthesia in case the brain expanded rapidly or if hemostasis was difficult to achieve during endoscopy. With the anesthesiologist on call, neurosurgeons intravenously administered pentazocine (7.5–15 mg) and diazepam (5–10 mg) for sedation depending on the patients’ consciousness. The patient’s head was placed on the horseshoe headrest followed by subcutaneous injection of 20 mL, 1% lidocaine with adrenaline (1:200,000). We simultaneously injected nicardipine to control the blood pressure when necessary. A 3 cm skin incision and burr hole were made according to the position of the hematoma on the CT scan with the help of the electromagnetic neuronavigation (StealthStation; Medtronic, Minneapolis, Minnesota, USA). The electromagnetic neuronavigation allows for frameless, pinless image-guided endoscopic hematoma removal^31^ under local anesthesia. The burr hole for basal ganglia hemorrhages was made by identifying the location where the distance from the burr hole to the hematoma was shortest and where the hematoma puncture was performed along the long axis of the hematoma. However, we avoided eloquent areas, including the pyramidal tract and language cortex, when making the tract. The burr hole for lobar hemorrhages was made over the hematoma where the lesion was closest to the surface. After a cruciate dural incision, we inserted the ventricular puncture needle into the hematoma under the navigation guide. We confirmed that we reached the hematoma by aspirating it through the ventricular puncture needle; then, we dilated the tract using a 10 Fr soft catheter insertion. A regular-type (diameter of 10 mm) or mini-type (6.8 mm) transparent sheath, (Neuroport; Olympus, Tokyo, Japan) was inserted along the tract to create the operating space for the neuroendoscope. A neurosurgeon introduced a rigid endoscope (Hopkins II, 2.7 mm, 0° angle; Karl Storz, Tuttlingen, Germany) through the Neuroport and used the combined irrigation-coagulation suction cannula (Fujita Medical Instrument, Tokyo, Japan), which can also be simultaneously used for irrigation and monopolar coagulation at its tip^24^ (Fig. 1) At times, another neurosurgeon checked the orientation using a neuronavigation pointer and endoscopic monitor. They also monitored the patients’ vital signs to ensure the safety of endoscopic manipulation and prepare for conversion to craniotomy under general anesthesia if necessary. When the patient was about to move, the endoscope and suction cannula were removed from the burr hole and additional pentazocine or diazepam was administered. We regarded the stiff portion of the hematoma as a bleeding point; therefore, we coagulated it firmly instead of attempting to remove it to avoid further damage to the white matter by rebleeding or manipulation. We also refrained from an aggressive hematoma removal near the internal capsule to preserve the pyramidal tract, which was not destroyed by hemorrhage, with the support of neuronavigation (intentional hematoma leaving technique). After hematoma removal, the burr hole was covered with the burr hole cover and the skin was sutured.

## Craniotomy procedure

Under general anesthesia, an adequate linear or small curved skin incision was made according to the preoperative CT scan. After removing the bone flap and opening the dura, we gained access to the hematoma cavity by corticotomy on the gyrus over the lobar hematoma, or on the temporal lobe or the insular cortex for basal ganglia hemorrhage. The hematoma was removed as much as possible under microscopic manipulation. The bleeding point was coagulated using bipolar electrocauteries. After sufficient decompression and confirmation of hemostasis, the removal cavity was filled with artificial cerebrospinal fluid. The dura mater was sutured and covered with oxidized cellulose. The piece of the cranium was fixed with metal plates, and the skin was sutured.

## Clinical variables

We collected data, including the physiological symptoms and medical history at admission, age, sex, hematoma location (basal ganglia or subcortex), GCS scores, National Institutes of Health Stroke Scale (NIHSS) scores, systolic blood pressure at admission, presence of habitual smoking, heavy drinking (over 450 g ethanol intake/week), comorbidities (history or present treatment by a clinician for hypertension, dyslipidemia, diabetes mellitus, cardiovascular diseases, stroke, chronic kidney diseases, or cancer), use of antithrombotic drugs (antiplatelet or anticoagulant), and operative time. The operative time was defined as the procedural time from the skin incision to the final suture. Application or non-application of the intentional hematoma leaving technique was confirmed from the operation records.

To evaluate the therapeutic effects of the operations, GCS scores on day seven and modified Rankin Scale (mRS) scores at 6 months after the operations of all 134 patients were collected by either personal outpatient interviews, reports from the rehabilitation hospital or home doctor, or interviews over the telephone, once the ethical approval was obtained for the study. The mRS score of 0–3 was considered as a favorable outcome. Rebleeding, hospital mortality, and other complications, such as infectious diseases, pulmonary embolism, heart failure, brain infarction, and disseminated intravascular coagulation, were also collected.

We measured the hematoma volume and the removal rate of the hematoma from the head CT on admission and just after the operation. The hematoma volume was calculated using the ABC/2 method^32^. Additionally, we investigated the presence of obvious destruction of the pyramidal tract on the CT image. We observed the primary motor area, radiate corona, posterior limb of the internal capsule, and cerebral peduncle for potential destruction. The obvious destruction of these areas indicated that the pyramidal tract was apparently destroyed; while the equivocal one was absent. Moreover, the temporal muscle area was measured on the head CT at admission as an indicator of sarcopenia^33^ and nutrition^34^ using the method reported by Katsuki et al.^35,36^.

## Statistical analysis

Results are presented as median (interquartile range). We investigated the before mentioned clinical variables for all 134 ICH patients, using the Mann–Whitney *U* test and the Fisher’s exact test; comparing the endoscopy and craniotomy groups. The Fisher’s exact test was used to investigate the association between the favorable outcome (mRS score 0–3) at 6 months and the surgical procedure, after adjusting for the stratified categorical data (age, hematoma location, use of antithrombotic drugs, GCS score severity, the distraction of pyramidal tract on the head CT image, and hematoma volume). We used Spearman’s rank correlation coefficient to investigate each variable. We then investigated the utility of the endoscopic procedures by conducting an ordinal logistic regression analysis and multiple regression analysis to adjust for the confounders. Potential factors with a *p* value of less than 0.10 and |r|> 0.20 in the univariate analysis were used for the ordinal logistic regression analysis and multiple regression analysis. Age was used for the ordinal logistic regression analyses as it is one of the known prognostic factors^37^. The adjusted odds ratio was computed by the exponentiation of the coefficient (labeled as B in Tables 3, 4, 5) calculated by the ordinal logistic regression analysis. We conducted these analyses using the SPSS software, version 24.0.0 of. (IBM, NY, USA). A two-tailed *p* < 0.05 was considered statistically significant.

## Results

### General characteristics

**Figure 2:**
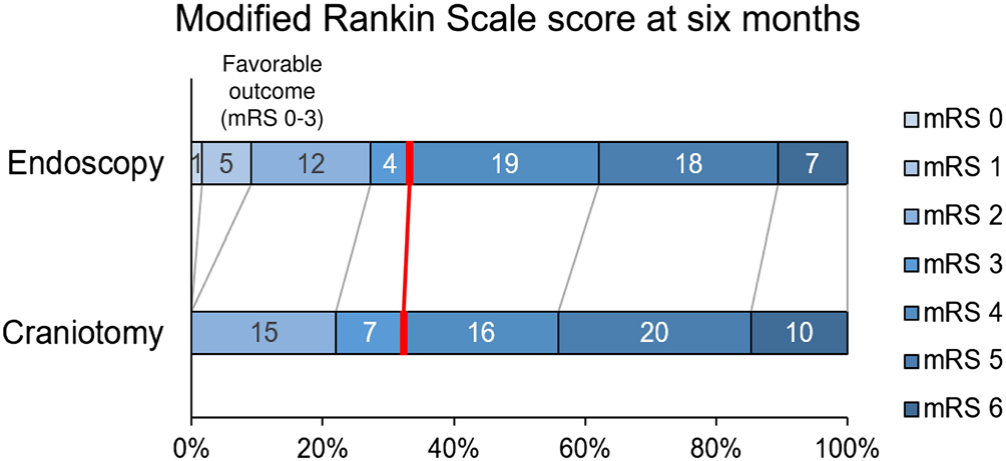
Modified Rankin Scale (mRS) score at 6 months after the surgery. Redline marks the division between the mRS scores 0–3 and 4–6. There was no significant difference between the mRS scores among the endoscopy and craniotomy groups at 6 months (Mann–Whitney *U* test; *p* = 0.29). No significant difference was observed in the distributions of the favorable and poor outcome favoring the endoscopy (Fisher’s exact test; *p* = 0.95, unadjusted odds ratio 0.957; 95% CI 0.465–1.971). The ordinal shift analysis using the ordinal logistic regression, after adjusting for age, hematoma location, Glasgow Coma Scale score on admission, history of stroke, and preoperative hematoma volume, did not reveal a significant shift of the mRS score at 6 months toward survival and independence by the endoscopic procedure (*p* = 0.331, adjusted odds ratio 1.493; 95% CI 0.664–3.353).

Table 2 shows the characteristics of the 134 patients (56 women and 78 men) with supratentorial ICH who were included in this study. The median age of the patients was 73 (61–82) years. Sixty-six patients (49%) underwent endoscopic hematoma removal. The median GCS and NIHSS scores at admission were 10 (7–13) and 23 (13–34), respectively. Ninety-five patients (71%) presented apparent destruction of the pyramidal tract on CT. The median GCS score on day seven was 13 (10–14) and the median mRS score at 6 months after the operation was 4 (3–5). Figure 2 shows the detailed mRS scores at 6 months for both the endoscopy and craniotomy groups. None of the patients required conversion to craniotomy during endoscopic hematoma removal. We were not intraoperatively disturbed by the patients’ body movements, change in the vital signs, or worsening of the respiratory conditions under local anesthesia. Other variables and the differences between the endoscopy and craniotomy groups are summarized in Table 2.

**Table 2:** Clinical characteristics of patients with supratentorial cerebral hemorrhage.

	All group (n = 134)	Endoscopy (n = 66)	Craniotomy (n = 68)	*p* value
Age (years)	73 (61–82)	73 (67–84)	73 (59–80)	0.40
36–65	n = 44	n = 15	n = 29	
66–75	n = 31	n = 21	n = 10	
76–85	n = 39	n = 19	n = 20	
86–95	n = 20	n = 11	n = 9	
Women:men (%women)	56:78 (42%)	30:36 (45%)	26:42 (38%)	0.39
Hematoma location, no. (%)				
Basal ganglia	78 (58%)	41 (62%)	37 (54%)	0.39
Subcortex	56 (42%)	25 (38%)	31 (46%)	
Apparent destruction of pyramidal tract on CT	95 (71%)	48 (73%)	47 (69%)	0.57
GCS score on admission	10 (7–13)	10 (8–13)	9 (7–13)	0.14
NIHSS score at admission	23 (13–34) (n = 78)	23 (13–27) (n = 60)	18 (17–40) (n = 18)	0.17
Systolic blood pressure (mmHg)	166 (143–188)	169 (136–188)	171 (146–189)	0.84
Smoking, no. (%)	39/96 (41%)	26/66 (39%)	13/30 (43%)	0.82
Heavy drinking, no. (%)	21/98 (21%)	16/66 (24%)	5/32 (16%)	0.43
Hypertension, no. (%)	112 (84%)	58 (88%)	54 (79%)	0.47
Dyslipidemia, no. (%)	45 (34%)	32 (48%)	13 (19%)	0.001*
Diabetes mellitus, no. (%)	22 (16%)	16 (24%)	6 (9%)	0.02*
Use of antiplatelet drugs, no. (%)	25 (19%)	13 (20%)	12 (18%)	0.83
Use of anticoagulant drugs, no. (%)	22 (16%)	13 (20%)	9 (13%)	0.34
History of cardiovascular diseases, no. (%)	34 (25%)	19 (29%)	15 (22%)	0.43
History of stroke, no. (%)	28 (21%)	15 (23%)	13 (19%)	0.67
History of hepatic diseases, no. (%)	8 (6%)	4 (6%)	4 (6%)	0.99
History of chronic kidney diseases, no. (%)	11 (8%)	7 (11%)	4 (6%)	0.36
History of cancer, no. (%)	14 (10%)	6 (9%)	8 (12%)	0.78
Temporal muscle area (mm^2^)	324 (196–438)	295 (191–423)	295 (201–442)	0.51
Hematoma volume preop. (mL)	120 (70–163)	104 (65–138)	120 (83–181)	0.45
Hematoma removal rate	99 (85–100)%	98 (84–100)%	98 (90–100)%	0.30
Operative time (min)	88 (59–112)	63 (50–76)	105 (78–148)	< 0.001*
GCS score 1 week postop	13 (10–14), 6 died	13 (12–14), 2 died	12 (9–14), 4 died	0.22
mRS 6 months postop	4 (3–5)	4 (2–5)	4 (3–5)	0.29
mRS 0	1 (1%)	1 (2%)	0 (0%)	
mRS 1	6 (4%)	5 (8%)	0 (0%)	
mRS 2	17 (13%)	12 (18%)	15 (22%)	
mRS 3	21 (16%)	4 (6%)	7 (10%)	
mRS 4	35 (25%)	19 (29%)	16 (24%)	
mRS 5	38 (28%)	18 (27%)	20 (29%)	
mRS 6	17 (13%)	7 (10%)	10 (15%)	
Rebleeding, no. (%)	10 (7%)	4 (6%)	6 (9%)	0.75
Other complications, no. (%)	44 (33%)	19 (29%)	25 (37%)	0.37

Results are shown by the median (interquartile range).

*CT* computed tomography, *GCS* Glasgow Coma Scale, *mRS* modified Rankin Scale, *NIHSS* National Institutes of Health Stroke Scale*.*

*p < 0.05 by Mann–Whitney *U* test or Fisher’s exact test.

## Application of the intentional hematoma leaving technique

We retrospectively investigated the operation records of the 66 patients treated by endoscopy. Of them, 12 (18%) patients were subjected to the intentional hematoma leaving technique due to stiff hematoma to preserve the pyramidal tract near the hematoma. The median left hematoma volume was 4 mL (2–14 mL). Ten (83%) of the 12 patients had favorable outcomes.

## Differences between patients who underwent endoscopic hematoma removal and craniotomy

There were no significant differences in baseline characteristics between the endoscopy group and the craniotomy group, except for the presence of dyslipidemia and diabetes mellitus. Operative time was significantly shorter in the endoscopy group than that in the craniotomy group (*p* < 0.001). However, the median GCS score on day seven, median mRS score at 6 months after the operation, death during hospitalization, rebleeding, and other complications were not statistically different between the two groups (*p* = 0.22, 0.29, 0.99, 0.75, and 0.37, respectively) (Table 2).

**Figure 3:**
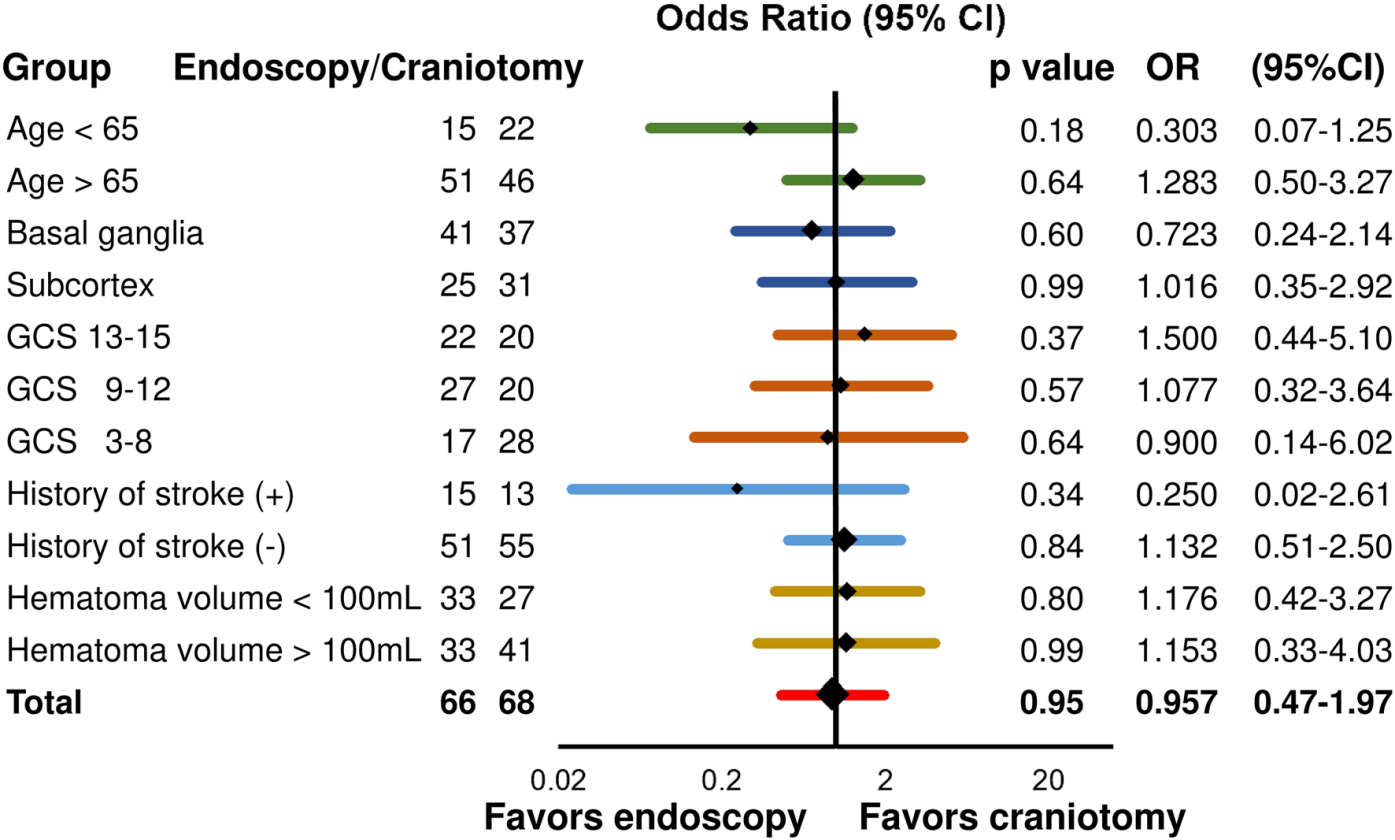
Shift towards the favorable outcome (modified Rankin Scale score 0–3) at 6 months adjusted for age, hematoma location, Glasgow Coma Scale (GCS) score severity on admission, the history of stroke, and the hematoma volume. The *p*-value, odds ratio (OR), and the 95% confidence interval (CI) were calculated using Fisher’s exact test.

The endoscopic procedure was not related to the favorable outcome. (Fisher’s exact test; *p* = 0.95, unadjusted odds ratio 0.957; 95% CI 0.465–1.971) (Figs. 2 and 3). After adjusting for the stratified categorical variables extracted from the univariate analysis (age, hematoma location, use of antithrombotic drugs, GCS score severity, history of stroke, and the hematoma volume; described in detail later with Table 4), the endoscopic procedure was not associated with the favorable outcome (Fig. 3). The cut-off of age was 65, according to the retirement age in Japan. That of hematoma volume was 100 mL based on the clinical experience. GCS score was divided into 3–8, 9–12, and 13–15^38^.

The GCS score on day seven was found correlated with the systolic blood pressure, GCS score on admission, and the preoperative hematoma volume (Spearman’s rank correlation coefficient, r = 0.252; *p* = 0.004; r = 0.716, *p* < 0.001; r = − 0.385, *p* < 0.001, respectively). Endoscopic procedure and hematoma at basal ganglia were associated with the GCS score on day seven (Mann–Whitney *U* test; *p* = 0.051 and 0.008, respectively). Using these potential factors in addition to age (r = − 0.073, *p* = 0.411), the ordinal logistic regression analysis revealed that age (*p* = 0.028, adjusted odds ratio 0.970; 95% CI 0.953–0.997) and GCS score on admission (*p* < 0.001, adjusted odds ratio 1.799; 95% CI 1.543–2.094) were associated with the GCS score on day seven. Endoscopic procedure was not associated with the GCS score on day seven (*p* = 0.079, adjusted odds ratio 0.552; 95% CI 0.284–1.071) (Table 3).

**Table 3:** Shift analysis using the ordinal logistic regression analysis for factors associated with the GCS score on day seven.

Factor	B	Standard error	*p* value	95% confidence interval for B
Age	− 0.03	0.014	0.028*	− 0.048 to − 0.003
Hematoma at basal ganglia	0.404	0.369	0.273	− 0.319 to 1.127
GCS score on admission	0.587	0.078	< 0.001*	0.434 to 0.739
Systolic blood pressure	0.002	0.005	0.642	− 0.007 to 0.011
Hematoma volume preop	− 0.002	0.003	0.536	− 0.007 to 0.004
Use of endoscope	− 0.595	0.339	0.079	− 1.259 to 0.069

Ordinal logistic regression analysis was performed using, in addition to age, the factors selected by univariate analysis with values of *p* < 0.10 and |r|> 0.20; hematoma at basal ganglia, GCS score and systolic blood pressure on admission, preoperative hematoma volume, and the use of the endoscope.

B, coefficient; GCS, Glasgow Coma Scale.

*p < 0.05 by ordinal logistic regression analysis.

The mRS score at 6 months correlated with age, GCS score on admission, and preoperative hematoma volume (Spearman’s rank correlation coefficient; r = 0.271, *p* = 0.002; r = − 0.415, *p* < 0.001; r = 0.371, *p* < 0.001). The endoscopic procedure was not associated with the mRS score at 6 months (Mann–Whitney *U* test; *p* = 0.349). History of stroke and hematoma at basal ganglia were associated with the mRS score at 6 months (Mann–Whitney *U* test; *p* =  0.081, 0.011). Using these potential factors in addition to the use of the endoscope, we performed an ordinal logistic regression analysis. We observed that age (*p* < 0.001, adjusted odds ratio 1.055; 95% CI 1.028–1.084), GCS score on admission (*p* = 0.001, adjusted odds ratio 0.832; 95% CI 0.742–0.931), and hematoma at basal ganglia (*p* = 0.003, adjusted odds ratio 0.329; 95% CI 0.156–0.692) were associated with the mRS score at 6 months. Endoscopic procedure was found not associated with mRS at 6 months (*p* = 0.250, adjusted odds ratio, 1.454; 95% CI 0.767–2.759) (Table 4).

**Table 4:** Shift analysis using ordinal logistic regression analysis for factors associated with mRS score at 6 months.

Factor	B	Standard error	*p* value	95% confidence interval for B
Age	0.054	0.014	< 0.001*	0.027 to 0.081
Hematoma at basal ganglia	− 1.110	0.379	0.003*	− 1.852 to − 0.368
GCS score on admission	− 0.184	0.058	0.001*	− 0.298 to − 0.071
History of stroke	− 0.296	0.409	0.470	− 1.097 to 0.506
Hematoma volume preop	0.005	0.003	0.050	− 0.00001 to 0.010
Use of endoscope	0.375	0.326	0.250	− 0.264 to 1.015

Ordinal logistic regression analysis was performed using, in addition to the use of the endoscope, the factors selected by univariate analysis with *p* < 0.10 and |r|> 0.20; Age, hematoma at basal ganglia, GCS score, history of stroke, and preoperative hematoma volume.

B, coefficient; GCS, Glasgow Coma Scale; mRS, modified Rankin Scale.

*p < 0.05 by ordinal logistic regression analysis.

The operative time correlated with the GCS score on admission and the preoperative hematoma volume (Spearman’s rank correlation coefficient, r = − 0.256, *p* = 0.003; r = 0.279, *p* = 0.001, respectively). The presence of dyslipidemia and cardiovascular disease was also related to the operative time (Mann–Whitney *U* test; *p* = 0.05 and 0.09, respectively) Using these potential factors, we performed a multiple regression analysis and observed that the use of endoscopy and preoperative hematoma volume were the factors independently associated with the operative time (*p* < 0.001 and *p* = 0.02, respectively) (Table 5).

**Table 5:** Multiple regression analysis for factors associated with operative time.

Factor	B	Standard error	Beta	t	*p* value	95% confidence interval for B
Use of endoscope	− 45.1	6.98	− 0.493	− 6.5	< 0.001*	− 58.9 to − 31.3
Presence of dyslipidemia	− 6.0	7.33	− 0.061	− 0.8	0.42	− 20.4 to 8.5
History of cardiovascular diseases	− 13.0	7.56	− 0.123	− 1.7	0.09	− 27.9 to 1.9
Hematoma volume preop	0.113	0.05	0.172	2.4	0.02*	0.02 to 0.20

Multiple regression analysis was performed using the factors selected by univariate analysis with *p* < 0.10 and |r|> 0.20; use of the endoscope, presence of dyslipidemia, history of cardiovascular disease, and preoperative hematoma volume.

B, coefficient.

*p < 0.05 by multiple regression analysis.

## Discussion

We compared the utility of endoscopic hematoma removal with that of craniotomy for ICH. There were no differences in the GCS scores at postoperative day seven nor the mRS scores at 6 months between the endoscopy and craniotomy. However, our study showed that endoscopic surgery significantly reduces the operative time compared to craniotomy.

## Operative time reduction by endoscopic surgery under local anesthesia

The operative time reduction is favorable under limited medical resources. Moreover, our procedure under local anesthesia further contributed to the time reduction until the operation, in addition to avoiding the side effect of general anesthesia. This is advantageous for the very neurologically severe patients with massive ICH, which require an immediate decrease of the intracranial pressure and for those with comorbidities that restricts the usage of general anesthesia. In fact, endoscopic surgery reportedly reduces complications compared to decompressive craniectomy surgery for putaminal hemorrhages of more than 50 mL^39^. Moreover, as a further possibility, endoscopy could also be used for patients that require immediate intracranial pressure reduction due to a hemorrhagic infarction^40^.

## Endoscopy compared with craniotomy

Table 1 summarizes the previously reported three meta-analyses that compared the endoscopic hematoma removal with craniotomy^26-28^. Scaggiante^28^ compared the combination of endoscopy and the stereotactic hematoma evacuation as a minimally invasive surgery option to craniotomy, or endoscopy alone to both the craniotomy and medication. Therefore, a direct comparison of our results to Scaggiante’s study is difficult; however, our results report a slightly better mortality rate. Zhao and Nam^26,27^ conducted the meta-analyses using the same three randomized controlled trials and concluded that the endoscopic procedures reduce complications. However, we did not observe a similar reduction in complications in our results. This may be because the ages of our patients were higher than those in the two reports. Finally, the previous two meta-analyses did not show an improvement in the functional outcome or mortality similar to our results. Further prospective studies are required to clarify which patients will benefit from an improved functional outcome by endoscopic hematoma removal.

Apart from the meta-analyses, seven prospective and retrospective reports concluded that the chronic ADL scores improved following endoscopic hematoma removal compared to those following craniotomy^17–19,21–23,25^. However, these results were produced from selected patients, which does not fully reflect real clinical practice. For example, many studies excluded cases with comorbidities^19,20^ or severe neurological conditions, including patients with very low GCS scores^17,21,22,24^, patients using antithrombotic drugs^17,18^, and patients with massive hematomas (over 60 mL)^18,22^. Further, they included patients who did not represent clear surgical indications according to the Japanese Guidelines for the Management of Stroke 2015^29^ nor 2009^30^, such as a hematoma volume between 20 and 30 mL^17,23,24^. Unlike these previous reports, the majority of patients in our study were regarded as surgically indicated in real clinical routine work according to the guidelines; many of our patients had comorbidities and severe neurological conditions. Therefore, even though our study did not exhibit the advantages of the endoscopic procedure in short- and long-term outcomes, it was meaningful in terms of reflecting the real clinical setting. We believe the efficacy of endoscopy still exists and further randomized control trials are required to fully investigate its utility.

## Intentional hematoma leaving technique

We have two technical key points in our endoscopic procedures. First, we regarded the stiff portion of the hematoma as a bleeding point, and therefore, we coagulated it firmly instead of attempting to remove it. Second, we also refrained from an aggressive hematoma removal near the internal capsule to preserve the pyramidal tract, which was not destroyed by hemorrhage, with the support of neuronavigation. Phase III of the MISTIE trial showed improvements of 1-year outcomes in the sub-analyzed patients with an increased hematoma removal rate (≤ 15 mL residual hematoma after the surgery)^14^. However, 10 of the 12 patients subjected to the intentional hematoma leaving technique had favorable outcomes in our study and we herein propose this technique.

The most important aspect regarding the endoscopic procedure is hemostasis. Under the endoscopic field, the coagulator and its operability are limited. Uncertain hemostasis leads to a postoperative hemorrhage, resulting in deleterious outcomes^41^. Therefore, we regarded the stiff portion of the hematoma as a bleeding point and coagulated it firmly instead of attempting to remove it. In fact, our data showed that the postoperative rebleeding rate was slightly higher in the craniotomy group than that in the endoscopy group. We speculate why the hematoma near the bleeding point was hard as follows; The bleeding vessel would be enhanced by vessel wall imaging like a ruptured aneurysm^42^ or dissection^43^, which suggests local inflammation^44^. The local inflammation would increase the coagulation, which would lead to the hardness of the hematoma^45^. On the other hand, microglia in the super-acute phase of the ICH has a protective role as an M2-macrophage^46^. The M2-polarized macrophage has an anti-inflammatory effect and promotes hematoma resolution^46^, and would, therefore, act around the rest of the hematoma except near the bleeding point. We believe that these are the likely reasons for the local difference of the hematoma hardness due to the various inflammation degrees in the hematoma cavity. This hypothesis should be evaluated by basic research or another study on the operative findings.

The hematoma caused cerebral edema and it made it difficult to interpret the CT image on admission regarding whether the putaminal and lobar hematoma had completely destroyed the pyramidal tract or not. Besides, the primary motor area^47^, corona radiata^48^, and the posterior limb of the internal capsule^49^ have a subdivided organization of pyramidal tract fibers. Therefore, there is a possibility that the pyramidal tract was not completely destroyed by the ICH due to the anatomical and radiological reasons described above. We should refrain from an aggressive hematoma removal near the pyramidal tract under endoscopic conditions, under which ones fall into disorientation rather than a craniotomy, to save the pyramidal tracts not destroyed by hemorrhage. In summary, the concept of our intentional hematoma leaving technique is that, in order to preserve the pyramidal tract and to avoid further damage to the brain, one should refrain from an aggressive hematoma removal with support of neuronavigation, considering the hematoma stiffness. The only 12 patients were found subjected to this technique from the operation records; however, the exact number of patients and the left hematoma volume are unknown as we always performed the endoscopic surgery with the before mentioned qualities in mind and did not necessarily describe the application of the technique in the operation records.

## Limitations

The operators in our hospital are well-trained for endoscopic hematoma removal and craniotomy. However, we gradually transitioned from craniotomy to endoscopic hematoma removal as a first-choice treatment between 2014 and 2015. During this period, patients who received antithrombotic drugs and displayed apparent extravasation on the contrast-enhanced CT image were likely to undergo craniotomy. In contrast, patients who presented with severe neurological conditions, such as GCS scores 3–8, were more likely to undergo endoscopic hematoma removal than a craniotomy due to the urgency for rapid hematoma removal due to herniation. Moreover, both surgical methods were still performed after the transition period at weekends/holidays. In addition to this and due to the observational study design, strong confounding factors may exist resulting from the varying indications and propensities regarding the decision-making process for performing the endoscopy or craniotomy procedures. Our findings are preliminary; thus, further prospective studies are required with larger sample sizes.

## Conclusions

There were no differences in the complication and mortality rates during hospitalization, GCS scores on day seven, and mRS scores at 6 months after the operation among patients in the endoscopy and craniotomy group. However, the operative time for the endoscopic surgery was shorter than that of the craniotomy.

### Compliance with ethical standards

We obtained written informed consent for this study from all the patients, legally authorized representatives of the relevant patients, and next of kin of the deceased patients. The hospital ethics committee approved this study.
